# Immune-Related Adverse Events Associated with Atezolizumab: Insights from Real-World Pharmacovigilance Data

**DOI:** 10.3390/antib13030056

**Published:** 2024-07-15

**Authors:** Connor Frey, Mahyar Etminan

**Affiliations:** 1 Department of Medicine, University of British Columbia, 317–2194 Health Sciences Mall, Vancouver, BC V6T 1Z3, Canada; 2 Department of Ophthalmology and Visual Sciences, University of British Columbia, 2550 Willow Street, Vancouver, BC V5Z 3N9, Canada; etminanm@mail.ubc.ca

**Keywords:** atezolizumab, immune checkpoint inhibitors, immuno-oncology, immune-related adverse events, irAE, pharmacovigilance, FAERS

## Abstract

The advancement of immuno-oncology has brought about a significant shift in cancer treatment methods, with antibody-based immune checkpoint inhibitors like atezolizumab leading the way in this regard. However, the use of this checkpoint blockade can result in immune-related adverse events due to increased T-cell activity. The full spectrum of these events is not yet completely understood. In this study, the United States FDA Adverse Event Reporting System (FAERS) was utilized to investigate immune-related adverse events linked with the use of atezolizumab. The study identified forty-nine immune-related adverse events that affected multiple organ systems, including cardiovascular, respiratory, hematologic, hepatic, renal, gastrointestinal, neurologic, musculoskeletal, dermatologic, endocrine, and systemic disorders. The strongest signals for relative risk occurred for immune-mediated encephalitis (RR = 93.443), autoimmune myocarditis (RR = 56.641), immune-mediated hepatitis (RR = 49.062), immune-mediated nephritis (RR = 40.947), and autoimmune arthritis (RR = 39.382). Despite the morbidity associated with these adverse events, emerging evidence suggests potential associations with improved survival outcomes. Overall, this report sheds light on the widespread immune-related adverse events that cause significant morbidity and mortality in patients with cancer being treated with atezolizumab and brings attention to them for the clinicians treating these patients.

## 1. Introduction

The development of immuno-oncology modalities has changed the landscape of cancer treatment. One such group, the immune checkpoint inhibitors (ICIs), specifically atezolizumab, has revolutionized the treatment of various cancers and has been shown to improve overall and progression-free survival [1,2]. Atezolizumab is used in urothelial carcinoma for patients who cannot have cisplatin-containing chemotherapy and have PD-L1 expression on tumours, in non-small-cell lung cancer as adjuvant therapy after surgery and platinum-based chemotherapy, first-line treatment of metastatic non-small cell lung cancer with high PD-L1 expression, either alone or with bevacizumab, paclitaxel, and carboplatin. It is also used for extensive-stage small-cell lung cancer with carboplatin and etoposide, in hepatocellular carcinoma with bevacizumab for first-line treatment of unresectable or metastatic disease, and BRAF mutation-positive unresectable or metastatic melanoma in combination with cobimetinib and vemurafenib. Binding to Programmed death-ligand 1 (PD-L1) expressed on tumour cells blocks the ability to downregulate cluster of differentiation 8 (CD-8)+ T-cells, increasing the immunogenic activity within the tumour microenvironment, and leading to cytotoxic effects on tumour cells. However, it can be expected that increased T-cell activity will lead to a myriad of immune-related events that have not been fully elucidated following the induction of atezolizumab. Immune-related adverse events (irAEs) associated with ICIs are a significant concern, as they can affect any organ system, presenting as dermatologic, gastrointestinal, hepatic, endocrine, or pulmonary toxicities, among others [3]. These irAEs range from mild to severe and can significantly impact patient quality of life and treatment outcomes if not promptly recognized and managed. Interestingly, it has also been suggested that the presence of these irAEs correlates to a better outcome while on therapy [4]. Thus, in the interest of improving clinical outcomes and reducing morbidity, these irAEs ought to be understood. We sought to examine the irAEs following the use of atezolizumab using the United States Food and Drug Administration Adverse Event Reporting System (FAERS) to provide a guide to physicians prescribing this medication to be aware of these events, ultimately improving the treatment outcomes for patients with cancer.

## 2. Materials and Methods

FAERS systematically compiles reported adverse drug reactions associated with all drugs submitted to the FDA by pharmaceutical manufacturers, healthcare providers, and consumers within the United States. Additionally, it incorporates post-marketing clinical trial reports stemming from studies conducted both within the United States and internationally. Due to the public nature of the data, an ethics committee was not involved in this study. Disproportionality analyses involve the comparative assessment of the reported incidence of a specific irAE with a given drug against the background reported rate of irAEs associated with all other pharmaceuticals. Leveraging the AERSMine software (Version 2024-06-07) (Department of Biomedical Informatics, Cincinnati Children’s Hospital Medical Center, Cincinnati, USA), a search was executed for “atezolizumab” as the primary suspect drug with “immune system disorders” with search periods spanning from 2004 Q1 to 2023 Q3 to compile a catalogue of irAEs, collected on the 8 June 2024. The software incorporates rigorous data cleansing procedures, encompassing rectifying improperly formatted entries and consolidating diverse nomenclatures referring to the same drug under a single domain, and duplicate and disease-specific events were manually filtered. The study utilized AERSMines default settings with Benjamini and Hochberg correction for FDR, requiring a *p*-value threshold of <0.005, with criteria set at absolute counts ≥ 10, relative risk ≥ 2, and defining a safety signal as >0. To determine the relative risk (RR), we compared the rate of irAE in patients given atezolizumab to the rate of the same events for all other drugs. Higher relative risk values indicate a stronger correlation between a specific drug and irAE, and an RR greater than 2.00 is generally considered “clinically significant”, as the risk is then at least doubled. Additionally, we utilized the AERSMine safety signal (SS) to analyze the association between atezolizumab and a given irAE.

## 3. Results

**Figure 1:**
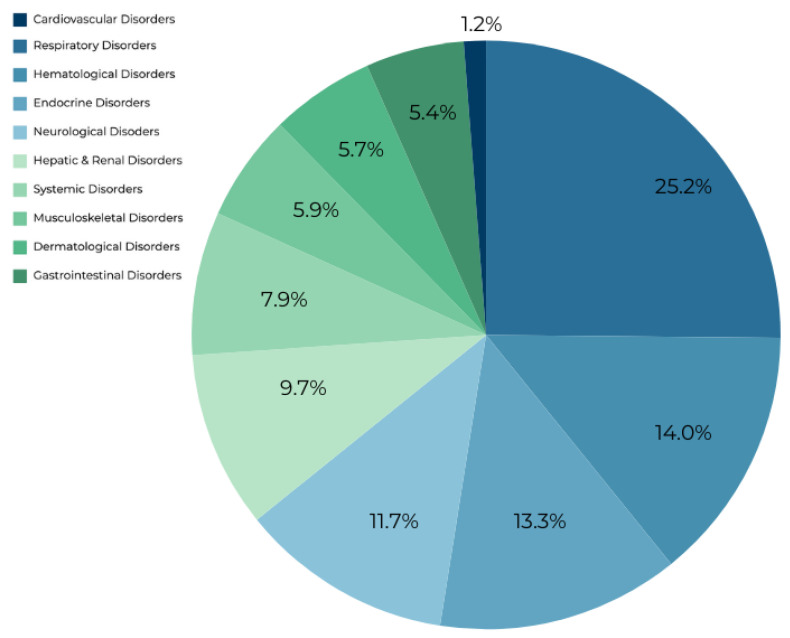
Breakdown of the percentage each organ system makes up of the irAEs associated with atezolizumab usage.

Forty-nine irAEs were reported, comprising a total of 455,949 unique individual reports, with 2958 of them associated with atezolizumab usage (Table 1, Figure 1). Notable findings included two cardiovascular and seven hematologic irAEs. Autoimmune myocarditis exhibited an SS of 8.992 and an RR of 56.641, while immune-mediated myocarditis had an SS of 7.052 and an RR of 13.733. Hemophagocytic lymphohistiocytosis demonstrated the highest SS among hematological disorders at 6.187 and an RR of 7.478. Similarly, autoimmune hemolytic anemia showed an SS of 5.876 and an RR of 5.995. Neutropenia, disseminated intravascular coagulation, lymphopenia, and immune thrombocytopenia exhibited SS values ranging from 3.218 to 4.797, with corresponding RRs ranging from 2.828 to 6.249. Respiratory disorders, representing 28.5% of reports, prominently featured immune-mediated pneumonitis with an SS of 8.064 and an RR of 28.403, followed closely by immune-mediated lung disease with an SS of 8.059 and an RR of 28.312. Interstitial lung disease, despite being the most frequently reported irAE with atezolizumab usage (*n* = 679), had a lower SS value of 6.072 and an RR of 6.881. Additionally, six hepatic and renal disorders were identified, with immune-mediated hepatitis and nephritis demonstrating significant SS values of 8.802 and 8.561, respectively, and corresponding RRs of 49.062 and 40.947. Autoimmune colitis and immune-mediated enterocolitis presented SS values of 8.056 and 7.947, with RRs of 28.249 and 26.099, respectively, while pancreatitis exhibited a lower SS of 1.544 and an RR of 3.169. Neurological irAEs featured immune-mediated encephalitis with the highest SS of 9.63 and an RR of 93.443, followed by autoimmune encephalitis with an SS of 8.025 and an RR of 27.625. Musculoskeletal and dermatological disorders revealed autoimmune arthritis with the most significant SS of 8.508 and an RR of 39.382 among musculoskeletal disorders, while vitiligo exhibited the highest SS of 6.307 and an RR of 8.114 among dermatological disorders. Endocrine and systemic conditions encompassed fulminant type 1 diabetes mellitus with an SS of 7.229 and an RR of 15.576 among endocrine disorders, and cytokine release syndrome with an SS of 5.996 and an RR of 6.521 among systemic disorders.

## 4. Discussion

Real-world data from the FAERS database in this study show that atezolizumab can cause extensive immune-mediated damage across various organ systems. A total of forty-nine immune-mediated irAEs were documented, with a substantial number of unique individual reports after atezolizumab usage, totalling 2958.

### 4.1. Cardiac, Respiratory, and Hematologic Disorders

Numerous reports of myocarditis following atezolizumab usage have been reported in the literature, and are known side effects of ICIs [5]. It generated a noteworthy RR in our study, both for reports of autoimmune myocarditis and immune-mediated myocarditis, with 56.641 and 13.733, respectively. A recent systematic analysis of ICI-associated myocarditis case reports found that in 53.8% of patients, their presentation was life-threatening, highlighting a need for awareness around the cardiotoxicity of ICIs, especially in a population with rising rates of cardiovascular disease [6]. Respiratory complications have long been known to occur in ICIs, and atezolizumab especially, in agreeance with our results, where it was the most prevalent irAE with an RR of 6.881 for interstitial lung disease [7]. Interestingly, it has been reported that patients on ICIs who develop interstitial lung disease have a higher overall response rate and longer progression-free survival [8]. In the POPLAR and OAK studies, pneumonitis was found to occur in 3% and 1% of patients respectively [9,10]. In an observational study, anti-PD-L1 immunotherapies were associated with neutropenia, autoimmune hemolytic anemia, and immune thrombocytopenia, as well as others with bicytopenias, showing congruency with the findings of our study, among multiple reports in the literature [11,12]. Moreover, serious irAEs such as disseminated intravascular coagulation were reported, and have been found to occur at low incidences, but high mortality in patients in ICIs [13]. Moreover, many reports of hemophagocytic lymphohistiocytosis were observed, though not well reported on. A case report observed hemophagocytic lymphohistiocytosis in a stage IV lung adenocarcinoma following initiation of atezolizumab, requiring intensive therapy before the patient ultimately passed [14].

### 4.2. Hepatic and Renal Disorders

Both hepatitis and nephritis are rare events with few reports following the usage of atezolizumab, and in vitro studies have shown atezolizumab to be hepatotoxic [15]. Our study generated an RR of 49.062 and 40.947 for immune-mediated hepatitis and nephritis, respectively, with an SS of 8.802 and 8.561. A review of renal toxicity following atezolizumab usage shows various types of immune-mediated nephritis occurring with its administration including tubulointerstitial nephritis, IgA glomerulonephritis, and membranoproliferative glomerulonephritis [16]. Cholangitis is frequently reported in the literature following atezolizumab usage and can be found co-occurring with secondary immune-mediated processes in abdominal organs such as gastritis and pancreatitis [17,18].

### 4.3. Gastrointestinal Disorders

Colitis is another common abdominal process that can occur during anti-PD-L1 therapy. However, it can be worrisome, as it might be mistaken for the symptoms caused by the disease itself, chemotherapy, or inflammatory bowel disease, which all could impact a patient with cancer, and it can be severe enough to require surgical intervention, thus requiring thorough investigation [19]. Autoimmune colitis had an RR of 28.249 in our study with an SS of 8.056. Colitis occurred in approximately 1–3% of patients across multiple trials [10,20]. Pancreatitis is a lesser reported irAE, with rates of pancreatic injury for atezolizumab reported at 1.62%, as well as in vitro evidence for pancreatic damage with PD-L1 blockade [21,22].

### 4.4. Neurological Disorders

Encephalitis had the highest RR of 93.443 and a significant SS of 9.63 in our study. This indicates that it is not only a severe adverse effect, but also one to be cautious of when prescribing atezolizumab. Various reports have been published on atezolizumab-induced encephalitis across different types of diseases, and it can manifest in multiple ways [23]. Neuropathy is a more common event following ICI, and the Impassion130 trial saw 6% of patients develop peripheral neuropathy, and a European pharmacovigilance study saw 52 reports of peripheral neuropathy following atezolizumab use over ten years [24,25]. A further case study described a patient who developed encephalitis and neuropathy following treatment with nivolumab and atezolizumab, with paresthesia persisting to the end of treatment [26]. Myasthenia gravis has been shown to occur following induction of atezolizumab, and a pharmacovigilance study found 78 cases, with 34% resulting in death [27]. Guillain–Barré syndrome is a rare outcome following ICI use, though a study from the FAERS database found that ICI monotherapy was associated with the condition, as well as myasthenia gravis, encephalitis, myelitis, meningitis, and neuropathy, which agrees with our findings [28].

### 4.5. Endocrine Disorders

Disorders of the endocrine system were prominent in this study. Hypophysitis is rare following anti-PD-L1 therapy, though reports exist and our RR was elevated [29,30]. Disorders of the thyroid are common following immunotherapy and have been found to show a favourable outcome in hepatocellular carcinoma following atezolizumab treatment [31]. The incidence of hyperthyroidism and hypothyroidism following anti-PD-L1 therapy is reported to be 0.7% and 3.8–5.5%, respectively, and multiple cases of thyroid disorder post-atezolizumab have been reported [32,33,34]. Type 1 diabetes mellitus is an underreported and serious irAE if untreated, leading some patients to present with diabetic ketoacidosis [35]. The vast array of cases highlights the possible need for measuring hemoglobin A1c in all patients being treated with atezolizumab, or ICI in general [36,37]. Lastly, adrenal insufficiency also appears to be underreported in the literature, though a two-patient case series reported that their patients experienced adrenocorticotropic hormone deficiency in the absence of other pituitary hormone anomalies, while a pharmacovigilance study reported another seven cases [38,39].

### 4.6. Musculoskeletal and Dermatological Disorders

Rheumatologic conditions have been shown to arise following ICI therapy, though our highest RR of 28.249 for autoimmune arthritis appears to be underreported, with one case report of polyarthritis, with tenosynovitis following atezolizumab, while a multicenter study found a myriad of inflammatory conditions in their ICI-treated population [40,41]. Muscle inflammation is prevalent following atezolizumab use, with one report of a patient developing both myositis and myocarditis [42]. Lastly, polymyalgia rheumatica was found in 12.12% of patients in a combined cohort study and 13.7% in a multicenter study for all patients on ICIs, with no specific numbers for atezolizumab specifically, and developed while on combination therapy with bevacizumab in a patient with hepatocellular carcinoma [40,43,44]. As for cutaneous manifestations, vitiligo is an uncommon presentation following ICIs; however, a case study was published which found that a patient with metastatic urothelial carcinoma treated with atezolizumab developed depigmentation after initiation, and interestingly, was found to have an extremely durable response, leading the group to suggest that cutaneous manifestations may improve outcome in ICI-treated patients, as was found with interstitial lung disease [8,45].

### 4.7. Systemic Disorders

Lastly are disorders that affect the body widely. A 2020 pharmacovigilance study on ICI-mediated cytokine release syndrome from the WHO Global Pharmacovigilance Database discovered 58 cases of the condition, of which 43 were associated with programmed cell death protein 1 (PD-1)/PD-L1 blockade [46]. A recent study of 2672 patients on ICI therapy found that approximately 1% of the population developed cytokine release syndrome, suggesting that rates are much higher than initially reported [47]. As previously mentioned, a further patient developed cytokine release syndrome with concomitant hemophagocytic lymphohistiocytosis [48]. Systemic inflammatory response syndrome and multiple organ dysfunction syndrome are both rare events with no reports of multiple organ dysfunction syndrome in the literature, and systemic inflammatory response syndrome is mentioned in a case report [49]. Lastly, sarcoidosis has been well characterized, with its histology indistinguishable from the classic form [50]. As has been mentioned throughout, multiple reports have found that irAEs associated with atezolizumab may be linked to improved survival, and while there is an impact on morbidity with these irAEs, it may be beneficial for survival [51,52].

### 4.8. Strengths and Limitations

Utilizing AERSMine and FAERS offers many significant strengths that contribute to the robustness and reliability of this study. FAERS provides an extensive dataset including reports from various sources, such as pharmaceutical manufacturers, healthcare providers, and consumers in the United States, as well as post-marketing clinical trial reports from domestic and international studies. The data have been thoroughly cleansed using AERSMine software (Version 2024-06-07) to ensure high quality by eliminating duplicate records, correcting improperly formatted entries, and consolidating diverse nomenclatures. This study covers adverse events across multiple organ systems, providing a comprehensive view of potential side effects and enabling healthcare providers to better anticipate and address these issues. FAERS’ public nature promotes transparency and facilitates independent verification and further research by other scientists and regulatory bodies. This openness supports ongoing pharmacovigilance efforts and contributes to the continuous improvement of drug safety monitoring. Additionally, the large volume of data and the rigorous analytical methods employed can uncover rare but serious adverse events, offering critical insights that may not be evident in smaller clinical trials. This ability to detect and analyze rare events is crucial for improving patient safety and informing regulatory decisions. Finally, the study aims to provide practical guidance to clinicians by offering a comprehensive overview of immune-related adverse events associated with atezolizumab.

However, this study does have some limitations. Firstly, there may be issues related to incomplete or selective reporting in these databases, which can impact the comprehensiveness and accuracy of the collected data. Reporting biases can cause an underestimation or overestimation of the incidence and severity of adverse events. Secondly, there are unaccounted confounding factors that could influence the results. These factors, such as patient demographics, concomitant medications, and underlying health conditions, may not be fully captured or controlled for in the databases. These confounders can make it difficult to interpret the data and may mask true associations between the drug and the adverse event. Additionally, the quality of data in FAERS can vary significantly. Differences in reporting practices among healthcare providers, variations in patient self-reporting, and the subjective nature of assessing adverse events can lead to variability in the quality of reports. Lastly, it is important to note that studies of this type do not imply causality. Despite these limitations, as the number of patients receiving atezolizumab increases, more data will become available. This expanded dataset will allow for more robust observations of trends in immune-related adverse events. Over time, the accumulation of data will provide a solid foundation for further epidemiological studies, helping researchers to better understand the safety profile of ICIs and develop strategies to mitigate associated risks.

## 5. Conclusions

This study sheds light on the various immune-related adverse events which can occur after undergoing treatment with atezolizumab. As per Table 1, all 49 investigated adverse events had an RR greater than 2.0, which is considered clinically significant. It is imperative to conduct further epidemiological studies to understand better and quantify these adverse events. Additionally, research is needed to explore the molecular mechanisms responsible for these events so that they can be accurately predicted and effectively managed. We hope that this manuscript provides prescribing clinicians with a comprehensive overview of the immune-related adverse events that patients taking atezolizumab may experience.

## Figures and Tables

**Table 1:** Number of reports for each immune-related adverse event and their associated relative risk and safety signal.

Adverse Event	Total Events for All Drugs (*n*)	Total Events for Atezolizumab (*n*)	Relative Risk	Safety Signal
**Cardiovascular and Hematological Disorders**				
Autoimmune Myocarditis	217	20	56.641	8.992
Immune-Mediated Myocarditis	666	16	13.733	7.052
Neutropenia	44,826	85	9.538	3.389
Hemophagocytic Lymphohistiocytosis	5001	126	7.478	6.187
Disseminated Intravascular Coagulation	18,995	53	6.249	3.946
Autoimmune Hemolytic Anemia	3190	48	5.995	5.876
Lymphopenia	23,746	40	4.307	3.218
Myelosuppression	14,473	11	4.289	1.792
Immune Thrombocytopenia	3270	52	2.828	4.797
**Respiratory Disorders**				
Immune-Mediated Pneumonitis	289	14	28.403	8.064
Immune-Mediated Lung Disease	767	51	28.312	8.059
Interstitial Lung Disease	55,736	679	6.881	6.072
**Hepatic and Renal Disorders**				
Immune-Mediated Hepatitis	1299	105	49.062	8.802
Immune-Mediated Nephritis	234	16	40.947	8.561
Autoimmune Nephritis	219	12	26.358	7.961
Sclerosing Cholangitis	2319	35	8.549	6.381
Autoimmune Hepatitis	7472	107	8.105	6.305
Cholangitis	6705	12	7.506	3.305
**Gastrointestinal Disorders**				
Autoimmune Colitis	747	36	28.249	8.056
Immune-Mediated Enterocolitis	2081	93	26.099	7.947
Pancreatitis	58,727	31	3.169	1.544
**Neurological Disorders**				
Immune-Mediated Encephalitis	237	34	93.443	9.63
Autoimmune Encephalitis	1081	51	27.625	8.025
Peripheral Motor Neuropathy	1657	10	23.945	5.059
Peripheral Sensory Neuropathy	6338	25	14.004	4.445
Myasthenia Gravis	6105	112	10.426	6.663
Guillain–Barré Syndrome	5233	82	8.881	6.435
Meningitis	7627	17	7.229	3.622
Transverse Myelitis	1543	15	5.477	5.747
**Musculoskeletal Disorders**				
Autoimmune Arthritis	182	12	39.382	8.508
Myositis	9521	74	17.857	5.424
Dermatomyositis	2660	31	6.579	6.008
Polymyalgia Rheumatica	3178	26	4.602	5.498
Polymyositis	2131	17	4.486	5.461
Immune-Mediated Myositis	1462	14	3.683	5.179
**Dermatological Disorders**				
Vitiligo	2023	29	8.114	6.307
Erythema Multiforme	10,627	106	5.621	5.784
Dermatitis	20,121	34	3.035	3.199
**Endocrine Disorders**				
Hypophysitis	2327	20	29.519	5.569
Fulminant Type 1 Diabetes Mellitus	1215	33	15.576	7.229
Thyroiditis	3124	19	13.452	5.07
Adrenal Insufficiency	12,160	63	10.38	4.832
Type 1 Diabetes Mellitus	6513	107	8.267	6.333
Hypothyroidism	36,687	117	6.469	4.139
Hyperthyroidism	15,845	35	6.409	3.609
**Systemic Disorders**				
Cytokine Release Syndrome	11,425	132	6.521	5.996
Systemic Inflammatory Response Syndrome	4520	33	4.103	5.333
Sarcoidosis	5643	29	2.882	4.827
Multiple Organ Dysfunction Syndrome	23,785	39	2.094	2.058
Total	455,949	2958		

## Data Availability

Data sharing is not provided for this article as all the data utilized were publicly accessed from AERSMine https://research.cchmc.org/aers/home (accessed on 8 June 2024) and are thus already free to access.

## Abbreviations

ICI	Immune Checkpoint Inhibitors
PD-L1	Programmed Death-Ligand 1
PD-1	Programmed Cell Death Protein 1
CD-8	Cluster of Differentiation 8
irAE	Immune-Related Adverse Event
FAERS	Food and Drug Administration Adverse Event Reporting System
RR	Relative Risk
SS	Safety Signal
